# Physical and mental health outcomes of COVID-19 induced delay in oncological care: A systematic review

**DOI:** 10.3389/fonc.2023.998940

**Published:** 2023-01-27

**Authors:** Ella D. van Vliet, Anouk H. Eijkelboom, Anoukh van Giessen, Sabine Siesling, G. Ardine de Wit

**Affiliations:** ^1^ Center for Health Protection, National Institute of Public Health and the Environment (RIVM), Bilthoven, Netherlands; ^2^ Department of Research and Development, Netherlands Comprehensive Cancer Organization (IKNL), Utrecht, Netherlands; ^3^ Department of Health Technology and Services Research, Technical Medical Centre, University of Twente, Enschede, Netherlands; ^4^ Center for Nutrition, Prevention and Healthcare, National Institute of Public Health and the Environment (RIVM), Bilthoven, Netherlands; ^5^ Department of Health Sciences, Faculty of Science, Vrije Universiteit Amsterdam & Amsterdam Health Research Institute, Amsterdam, Netherlands

**Keywords:** COVID-19, cancer, systematic review, delay, mental health, stage, survival, mortality

## Abstract

**Background:**

During the COVID-19 pandemic cancer patients might have experienced delays in screening, diagnosis and/or treatment. A systematic review was conducted to give an overview of the effects of COVID-19 induced delays in oncological care on the physical and mental health outcomes of cancer patients.

**Methods:**

MEDLINE and EMBASE databases were searched for articles on the effects of COVID-19 induced delays on physical and mental health outcomes.

**Results:**

Out of 1333 papers, eighteen observational, and twelve modelling studies were included. In approximately half of the studies, tumor stage distribution differed during the pandemic compared to before the pandemic. Modelling studies predicted that the estimated increase in the number of deaths ranged from -0.04 to 30%, and the estimated reduction in survival ranged from 0.4 to 35%. Varying results on the impact on mental health, e.g. anxiety and depression, were seen.

**Conclusions:**

Due to large methodological discrepancies between the studies and the varying results, the effect of COVID-19 induced delays on the physical and mental health outcomes of cancer patients remains uncertain. While modelling studies estimated an increase in mortality, observational studies suggest that mortality might not increase to a large extent. More longitudinal observational data from the pandemic period is needed for more conclusive results.

## 1 Introduction

The first COVID-19 cases were confirmed in December, 2019, in Wuhan, China (1). Thereafter, the virus quickly spread around the world. Many countries introduced social measures to reduce spreading of the virus. The World Health Organization for instance recommended to keep at least 1 meter (approximately 3.3 feet) distance from each other and to cancel social activities (2). Despite these recommendations, the number of hospitalized COVID-19 patients quickly increased in many countries at the start of the pandemic (3). This put an enormous pressure on the health care for non-COVID-19 patients. Specific measures in oncological care were therefore taken to 1) ensure safe and effective care for all cancer patients, 2) divert hospital resources and intensive care unit capacity towards COVID-19 patients, and 3) prevent infection of patients and health care staff. These last two measures led to the suspension of the national screening programs for breast, colorectal, and cervix cancer (4, 5). Moreover, all three measures caused the introduction of COVID-19 induced cancer-specific treatment guidelines. These guidelines recommended to alter, delay or cancel treatment, prerequisite this would not have an effect on the long-term outcomes of cancer patients (6). Both the suspension of the screening programs and the introduction of COVID-19 induced treatment guidelines resulted in a delay in screening, diagnosis, and treatment (5, 7–9).

Besides delays in diagnosis due to suspension of the screening programs, delays in diagnosis could also have occurred because patients with suspected cancer signs or symptoms delayed their visit to the general practitioner (GP) themselves. They might for instance have been concerned about contracting the virus and/or they did not want to overburden the health care system (10). In addition, patients experienced difficulties in gaining access to the GP at the start of the pandemic, which might also have led to a delay in diagnosis (11).

An emerging number of studies have investigated how delays in screening, diagnosis, and/or treatment have impacted the physical and mental health of cancer patients. However, a robust overview of the health effects of COVID-19 induced delays on cancer patients is lacking. Therefore, we conducted a systematic review to give a first overview of the physical and mental health outcomes of delays in oncological care due to the COVID-19 pandemic.

## 2 Methods

A systematic review of the physical and mental health outcomes of cancer patients affected by delay in cancer screening, diagnosis and/or treatment, due to the COVID-19 pandemic was conducted.

### 2.1 Search strategy

An extensive search strategy was developed in collaboration with a scientific librarian to retrieve relevant articles. During the COVID-19 pandemic, different terminology and synonyms were used. Therefore, literature was searched for the range of terms that were used to describe the topics of interest to ensure the capture of relevant articles. The applied search strategy included relevant oncological terms (e.g. cancer, oncology, neoplasm and carcinoma), and terms associated with delay (e.g. postponed, disrupted, lockdown and paused), and COVID-19 (e.g. corona and sars-cov-2). The searches were performed in the PubMed/MEDLINE and EMBASE databases on November 8th, 2021. Only studies that were published in English or Dutch between January 2020 - November 2021 (corresponding to the COVID-19 pandemic) were included. There was no restriction regarding status of publication, e.g. pre-print articles were acceptable. The full search strategy is listed in supplementary materials 1.

### 2.2 Study selection and extraction

Two independent researchers (EV and AE) screened the literature using Covidence (Veritas Health Innovation, Melbourne, Australia). The selection process consisted of two phases. In phase one all abstracts and titles were evaluated. The full text of the remaining eligible articles was then screened in phase two. Any conflicts between the reviewers were resolved by discussion. When no agreement could be made a third reviewer (AW) was solicited. Articles were included if they: 1) included information on cancer patients, 2) presented estimates of the effect of delay on health outcomes and related the delay to the COVID-19 pandemic. Relevant health outcomes had to be quantified with accepted metrics, e.g. survival or tumor staging, or measured with validated instruments, e.g. for mental health or quality of life. In order to get a general overview, to optimize comparability and to minimize the influence of COVID epicenters studies were excluded if they: 1) were single center studies, 2) included data only on patients who were not currently being treated for cancer (i.e. survivors, other diseases, etc.), and 3) were other types of articles than research papers (i.e. conference abstracts, protocols, reviews etc.). A uniform extraction template was used for data extraction. The following information was extracted from each study: the first author’s last name, country, study design, type of cancer, type of delay, types of health outcomes studied, study population and main results. All articles were extracted by two researchers and consensus was reached prior to the analysis. Several authors were contacted for further clarification or more detailed information. The most frequently reported health outcomes were selected for the synthesis of the results.

## 3 Results

### 3.1 Selected studies

**Figure 1:**
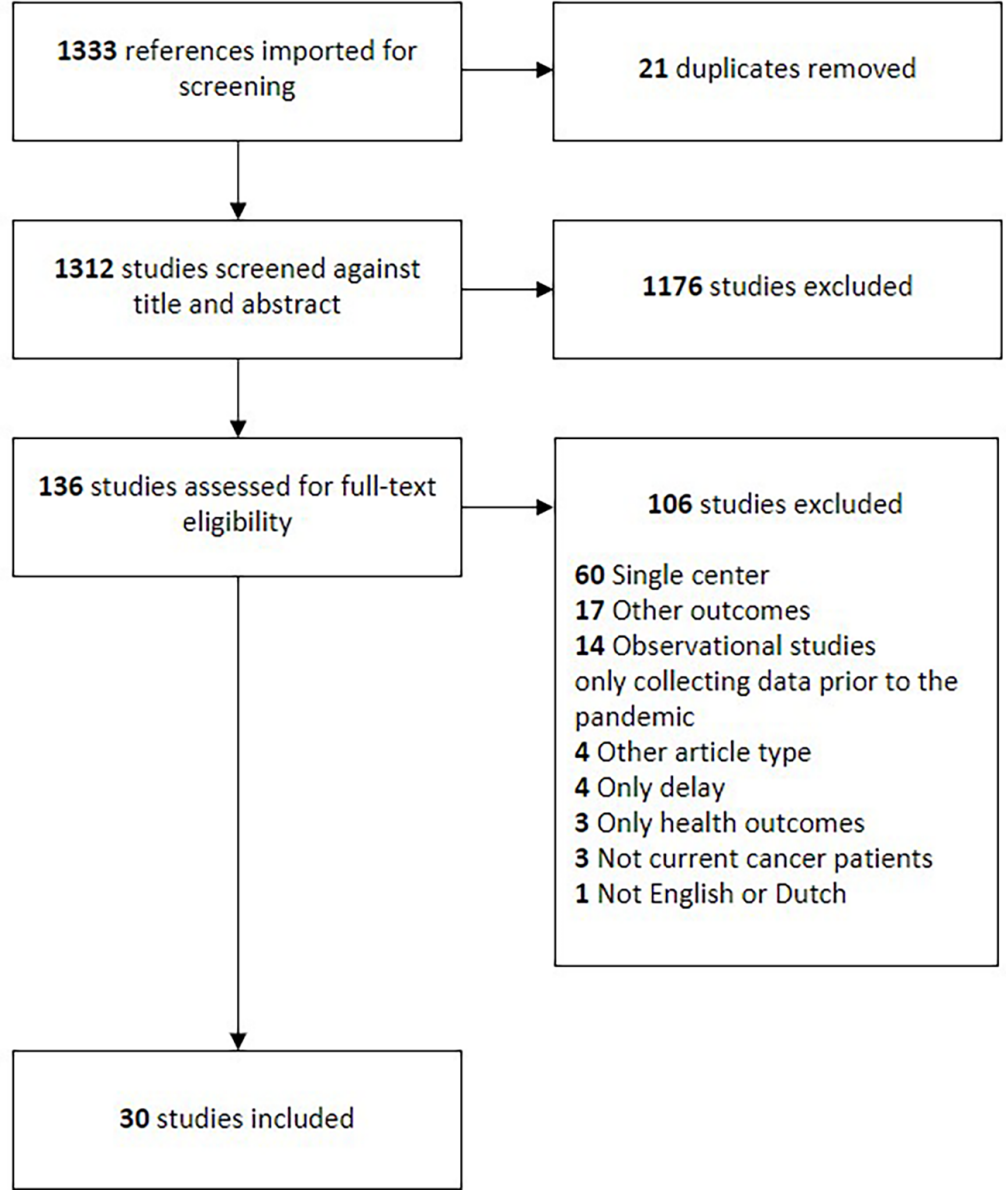
Flow chart of the screening process and the inclusion and exclusion results.

A total of 1333 studies resulted from the search strategy. Twenty-one studies were removed as duplicates. The remaining 1312 studies were screened on their titles and abstracts, after which 1176 articles were deemed irrelevant. This resulted in 136 articles for full-text assessment. One-hundred-and-six studies were excluded based on the full-text review, resulting in thirty articles included in the final analysis. The full flowchart of the screening process is shown in **Figure 1**.

**Figure 2:**
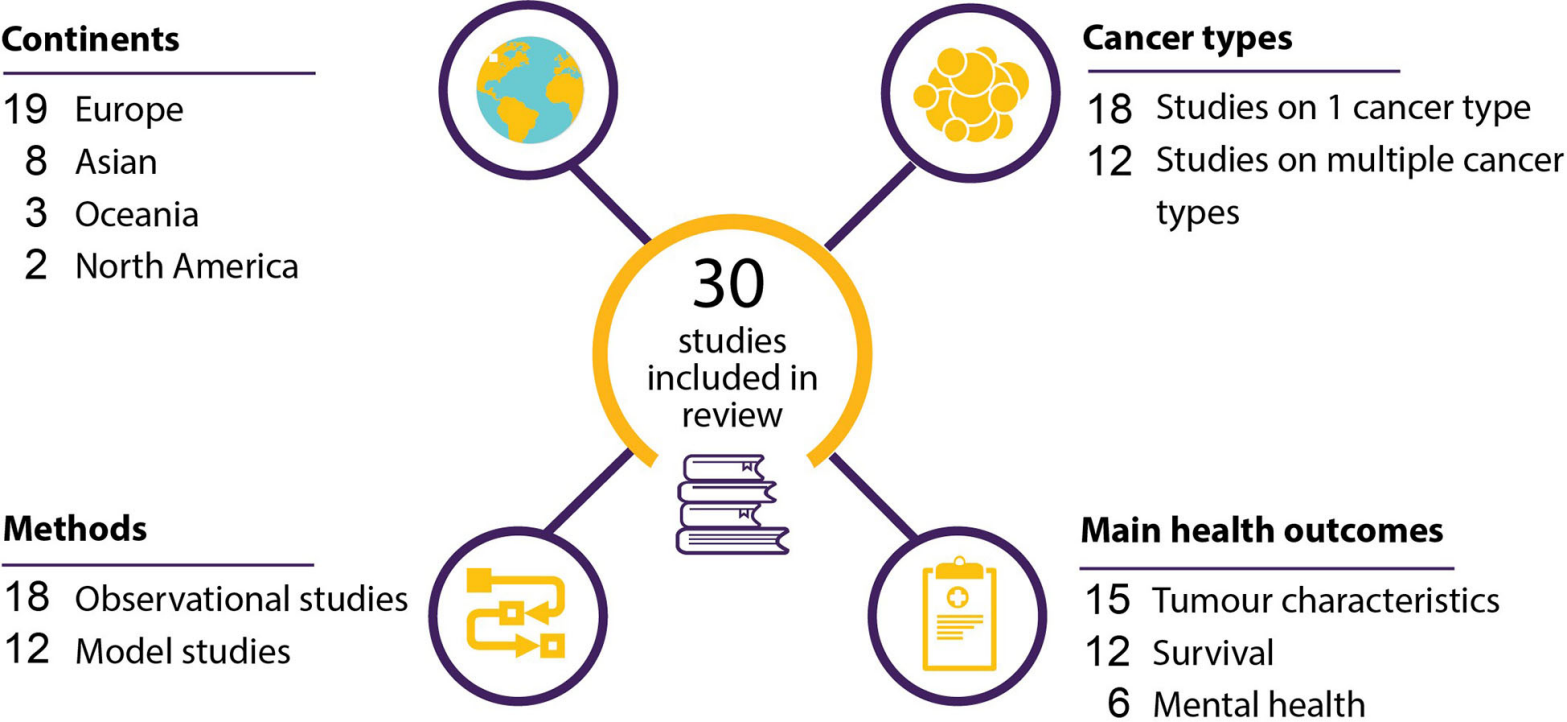
Overview of the characteristics of the included studies.

Twelve of the included studies compared the characteristics of tumors diagnosed during and before the pandemic (12–23), six were cross-sectional studies (24–29), and twelve were modelling studies (30–41). Nineteen of the studies were performed in Europe (12–16, 19–22, 24, 25, 31, 33, 36–41), three in Oceania (23, 31, 32), eight in Asia (17, 18, 26–29, 34, 35), and two in North-America (30, 31). The included studies investigated the effect of delays in oncological care on 36 different tumor types or groups of tumors. Tumor characteristics was the main health outcome in fifteen studies (**Table 1**) (12–23), survival in nine (**Tables 2**, **3**) (30, 32–34, 36–38, 40, 41), tumor characteristics and survival in three (31, 35, 39) and mental health in six (**Table 4**) (24–29). Results are discussed based on the type of health outcome studied: tumor characteristics, survival or mental health. The included studies are shown graphically in **Figure 2**.

**Table 1:** Effect of the Covid-19 induced delays on tumor characteristics.

Main author	Country	Cancer type(s)	Nation-wide population-based study	Study type	Control period(s) (N)	COVID-19 period(s) (N)	Did the tumor characteristics differ between the control and COVID-19 period?
**Aparicio** (12)	France	Digestive system	No, patients from 30 hospitals in the Greater Paris area	Observational	01-01 to 16-03 2018-2019 (1849), 17-03 to 10-05 2018-2019 (1295), 11-05 to 30-08 2018-2019 (2439)	01-01 to 16-03 2020 (pre-lockdown) (914), 17-03 to 10-05 2020 (lockdown) (373), 11-05 to 30-08 2020 (post-lockdown) (1012)	No, tumor stage distribution and 3-month overall survival did not differ between patients diagnosed during the pre-lockdown period of 2018, 2019, or 2020 (p=0.339 and p=0.89, respectively), the lockdown period of 2018, 2019, or 2020 (p=0.203 and p=0.57), or the post-lockdown period of 2018, 2019, or 2020 (p=0.493 and p=0.06).
**Blay (** 13 **)**	France	All	No, patients from 17 hospitals	Observational	January-July 2019 (47159)	January-July 2020 (43947)	Yes, higher proportion of stage IV tumors during COVID (22.2% vs. 24.3%, p<0.0001).
**Eijkel-boom** (14)	the Nether-lands	Breast	Yes	Observational	weeks 14-35 2018/2019 (average of 2018/2019: 4644)	weeks 14-35 2020 (2753)	Yes, tumor stage distribution differed significantly (p<0.05). After stratification by method of detection (screen- or non-screen-detected) tumor stage distribution did no longer differ.
**Gualdi** (15)	Italy	Cutaneous malignant melanoma	No, patients from 12 dermatologic institutions	Observational	01-05 to 31-07 2017-2019 (887)	01-05 to 31-07-2020 (237)	Yes, higher Brewslow thickness during COVID (0.5mm vs. 0.4mm).
**Heimes** (16)	Germany	Oral	No, patients from 3 hospitals	Observational	13-03 to 16-06 2018-2019 (160), 17-06 to 01-11 2018-2019 (278)	13-03 to 16-06 2020 (lockdown) (79), 17-06 to 01-11 2018-2020 (post-lockdown) (136)	Patients diagnosed post-lockdown had less often a stage III or IV tumor compared to patients diagnosed during the control period (50.8% vs. 59.2%). Tumor stage distribution did not differ between patients diagnosed during lockdown and the control period.
**Kuzuu** (17)	Japan	Gastrointestinal	No, patients from 2 hospitals	Observational	Jan 2017-Feb 2020 colorectal (1581), gastric (1164), pancreatic (532), esophageal (335), hepatocellular (338), or biliary tract (268)	Mar-Dec 2020 colorectal (360), gastric (224), pancreatic (141), esophageal (87), hepatocellular (75), or biliary tract (62)	Yes, significantly higher number of patients diagnosed per month with a stage III colorectal cancer during COVID (mean (SD): 12.10 (2.42) vs. 7.18 (2.85), p<0.001). For the other cancer types no increase was seen in the number of patients diagnosed with a stage III or IV tumor.
**Not, van** (20)	the Nether-lands	Irresectable stage IIIc or IV advanced melanoma	Yes	Observational	16-03 to 24-06 2018-2019 (339), 21-09 to 27-12 2018-2019 (455)	16-03-2020 to 24-06-2020 (first wave) (108), 21-09-2020 to 27-12-2020 (second wave) (166)	Yes, tumor stage distribution differed significantly between patients diagnosed during the second wave and the control period (p=0.001). Tumor stage distribution did not differ between patients diagnosed during the first wave and the control period (p=0.900).
**Park** (18)	South Korea	Lung, small and non-small cell	No, patients from 3 hospitals	Observational	Feb-Jun 2017-2019 non-small cell (386), and small cell lung (57)	Feb-Jun 2020 non-small cell (146) and small cell lung (23)	Yes, higher proportion (p=0.011) and a higher number (no p-value) of patients diagnosed with a stage III or IV non-small cell lung cancer during COVID (2017: 70 (57.9%), 2018: 82 (66.7%), 2019: 89 (62.7%), 2020: 109 (74.7%)). Tumor stage distribution did not differ for small-cell lung cancer (p=0.239).
**Purus-hotham** (19)	United Kingdom	Multiple (Breast, colorectal, prostate or lung)	No, patients diagnosed at the Guy’s & St Thomas’ NHS Trust hospitals	Observational	19-10-2019 to 20-03-2020 breast (233), colorectal (169), lung (238) and prostate (398)	20-04-2020 to 20-09-2020 breast (175), colorectal (77), lung (175), or prostate (132)	No, tumor stage distribution did not differ (p-values not shown).
**Vanni** (21)	Italy	Breast	No, patients from four hospitals	Observational	11-03-2019 to 30-05-2019 (209)	11-03-2020 to 30-05-2020 (223)	Yes, N-stage and grade distribution differed significantly (p=0.031 and p=0.032, respectively). More patients diagnosed with a N2 (16 (8.0%) vs. 4 (2.3%)), grade 2 (100 (49.2%) vs. 65 (37.8%)), or grade 3 tumor (52 (25.6%) vs. 36 (20.9%)) during COVID. Time between biopsy/cytological examination and surgery was significantly longer for patients diagnosed during COVID (42 (10-220) days vs. 56 (6-134) days, p<0.05). In the multivariable analysis, 10 extra days between biopsy/cytological examination and surgery was associated with an increased risk of lymph nodes involvement (OR: 1.07, 95% CI: 1.01-1.13).
**Wang** (22)	United Kingdom	Uveal melanoma	Yes	Observational	Mar-Jun 2018-2019 (556)	Mar-Jun 2020 (158), Jul-Oct 2020 (213)	Yes, higher proportion diagnosed with a stage III or IV tumor during July-October 2020 (28.2% vs. 13.4%, p=0.006). Tumor stage distribution did not differ between patients diagnosed in March-June 2018, 2019, or 2020.
**Williams** (23)	Australia and New Zealand	Colorectal	No, patients from surgeons volunteering in the BCCA registry	Observational	2^nd^ quartile (Apr-Jun) 2017-2019 (2889), and 4^th^ quartile (Oct-Dec) 2017-2019 (2910)	2^nd^ quartile 2020 (712), and 4^th^ quartile 2020 (324)	Yes, tumor stage distribution differed significantly between patients diagnosed in the 4^th^ quartile of 2017, 2018, 2019, or 2020 (p=0.017), with a higher proportion being diagnosed with a stage II (2017: 30.3%, 2018: 29.3%, 2019: 33.7%, 2020: 35.2%) or stage III tumor (2017: 29.0%, 2018: 33.6%, 2019: 29.4%, 2020: 34.3%) during COVID. No difference in proportion being diagnosed with a stage IV tumor (2017: 9.3%, 2019: 9.2%, 2019: 9.7%, 2020: 9.6%). Tumor stage distribution did not differ for patients diagnosed in the 2^nd^ quartile (p=0.202).
**Jen** (35)	Taiwan	Colorectal	Yes	Modelling	1) No delay, 2) 0.5-year delay in screening, 3) 1.0-year delay in screening, 4) 1.5-year delay in screening, 5) 2-year delay in screening		0.5-, 1.0-, 1.5-, and 2.0-years delay in screening increases the number of patients with a late-stage colorectal tumor by 25% (RR: 1.25, 95% CI: 1.18-1.33), 29% (RR: 1.29, 95% CI: 1.21-1.37), 34% (RR: 1.34, 95% CI: 1.26-1.42), and 39% (RR: 1.39, 95% CI: 1.31-1.48), respectively.
**Jonge, de** (31)	Australia, Canada, the Nether-lands	Colorectal	Yes	Modelling	1) No delay, 2) 6-month screening disruption, 3) 3-month screening disruption, 4) 12-month screening disruption, 5) 6-month screening disruption, 50% reduction in the first 3 months after screening restart, 25% reduction in the second 3 months after screening restart, 6) 6-month screening disruption, 50% reduction in the 6 months after screening restart, 7) 6-month screening disruption with immediate screening catch-up after restart, 8) 6-month screening disruption with delay catch-up screening		Depending on the type of disruptions it was predicted that in 2050 -0.5 to 0.5% more stage I tumors, 0.0 to 1.1% more stage II tumors, 0.0 to 2.0% more stage III tumors, and -0.2 to 5.9% more stage IV tumors would be diagnosed. A 6-month disruption was predicted to lead to -0.3 to 0.0% more stage I tumors, 0.2 to 0.6% more stage II tumors, 0.7 to 2.9% more stage III tumors, and 0.5 to 2.8% more stage IV tumors in 2030.
**Smith** (39)	Australia, the Nether-lands, Norway, US	Cervical	Yes	Modelling	1) No delay, 2) 12 months delay in primary screening, 3) 12 months delay in primary screening and surveillance, 4) 12 months delay in primary screening, surveillance and coloscopy/treatment		Depending on the type of disruptions it was predicted that during 2020-2030 0.0 to 1.2% of the tumors will be in a later stage.

CI, confidence interval; OR, odds ratio; RR, relative risk; SD, standard deviation.

**Table 2:** Effect of the Covid-19 induced delays on survival according to modelling studies.

Main author	Country	Cancer type(s)	Length of delay	Type of delay	Time horizon	Predicted effect on mortality/survival
**Alagoz** (30)	United States	Breast	6 months	Combination (screening, diagnosis and treatment)	10 years	0.52% (model range: 0.36 to 0.56%) extra deaths
**Degeling** (32)	Australia	Multiple (breast, colorectal, lung and melanoma)	3 and 6 months	Diagnosis	5 years	Excess mortality of 88 deaths after 5 years was estimated for a delay of 3 months, and 349 deaths for a delay of 6-months. This resulted in a range of 0.52-3.56% extra deaths.
**Gheorghe** (33)	United Kingdom	Multiple (breast, colon, rectum, esophagus and non-small cell lung)	12 months	Diagnosis	5 years	The number of total cancer deaths pre-pandemic were observed to be 44493. The predicted deaths after the first wave of the pandemic were 48109 (95% CI: 48020 – 48200). This resulted in 8.13% extra deaths
**Gupta** (34)	India	Cervical	9 weeks and 6 months	Combination (diagnosis and treatment)	lifetime	Over the lifetime of the cohort, an excess of cervical cancer deaths ranging from 795 (2.52%) to 2160 (3.80%) was estimated
**Jen** (35)	Taiwan	Colorectal	6 months, 1-year,1.5-years, and 2- years	Screening	15 years	A 26% (range: 21-32%) increase in the number of deaths after a 0.5-year delay, a 28% (range:22-33%) increase after a 1-year delay, a 29% (range: 24-34%) increase after a 1.5-year delay, and a 30% (25-36%) increase after a 2-years delay.
**Jonge, de** (31)	Multiple (Australia, Canada and the Netherlands)	Colorectal	6 months	Screening	30 years	With 6-month disruption and no catch-up screening or decrease in participation during the recovery period, 678 to 881 extra deaths were estimated for the Netherlands (0.4 to 0.6% relative increase), 1961 extra deaths were estimated for Australia (1.0% relative increase) and 1319 extra deaths were estimated for Canada (0.4% relative increase). With 6-month disruption and 50% reduction in participation during the recovery period 0.6 to 1.6% extra deaths were observed.
**Kregting** (36)	the Netherlands	Multiple (breast, cervical and colorectal)	3, 6 and 12 months	Screening	10 years	The cumulative breast cancer and cervical cancer mortality rates over the 10 years following the screening disruption (2020–2030) were the highest in the no catch-up strategy. The cumulative mortality rate was 2.0 per 100000 women for breast cancer (186 cases in the Dutch situation) and 0.3 per 100000 individuals for cervical cancer (27 cases in the Dutch situation). In colorectal cancer, the everyone delay strategy led to the highest cumulative mortality rate (4.9 per 100000; 740 cases in the Dutch situation). This resulted in a range of 0.04-3.08% extra deaths.
**Loveday** (37)	United Kingdom	Colorectal	2 and 6 months	Diagnosis	10 years	A 2 month delay in the diagnostic pathway is, depending on the age at diagnosis, predicted to result in a 0.4-7.5%, 4.9-8.2%, and 9.1-11.5% reduction in survival for patients with a stage I, stage II, or stage III tumor, respectively. A 6 month delay in the diagnostic pathway is, depending on the age at diagnosis, predicted to result in a 1.9-28.7%, 19.5-30.4%, and 29.7-35.0% reduction in survival for patients with a stage I, stage II, or stage III tumor, respectively.
**Maringe** (38)	United Kingdom	Multiple (breast, colorectal, esophagus and non-small cell lung)	12 months	Diagnosis	5 years	It is estimated that the delay in diagnosis will lead to 7.9-9.6% extra breast cancer-related deaths, 15.3-16.6% extra colorectal cancer-related deaths, 4.8-5.3% extra lung cancer-related deaths, and 5.8-6.0% extra esophageal cancer-related deaths, within 5 years.
**Smith** (39)	Multiple (Australia, the Netherlands, Norway and the United States)	Cervical	6 months	Screening	10 years	Additional deaths in the longer term resulting from these additional and upstaged cancer cases ranged from 0.0-16.6 per million women aged 20+.
**Sud** (41)	United Kingdom	Multiple (non-hematological malignancies)	3 and 6 months	Diagnosis	10 years	For several cancers, a 3-month delay in diagnosis is predicted to result in a reduction in long-term (10-year) survival of more than 10% in most age groups. Delays of 6 months are predicted to reduce 10-year survival by more than 30%.
**Sud** (40)	United Kingdom	Multiple (non-hematological malignancies)	3 and 6 months	Treatment	5 years	The greatest rates of deaths arise following even modest delays to surgery in aggressive cancers, with >30% reduction in survival at 6 months and >17% reduction in survival at 3 months.

CI, confidence interval.

**Table 3:** Effect of the Covid-19 induced delays on survival specified per type of delay.

		Screening	Diagnosis	Treatment	Combination
**2 months delay**	10 years		0.4-7.5%, 4.9-8.2%, and 9.1-11.5% reduction in survival for stage I, stage II, or stage III colorectal cancers (37)		
	life				2.52% extra cervical cancer deaths (34)
**3 months delay**	5 years		0.52% extra deaths of multiple cancer types (32)	>17% reduction in survival in multiple cancer types (40)	
	10 years	0.01-0.30% extra breast cancer, 0.03-0.61% extra cervical cancer, and -0.04-1.78% extra colorectal cancer deaths (36)	>10% reduction in survival in multiple cancer types (41)		
**6 months delay**	5 years		3.56% extra deaths of multiple cancer types (32)	>30% reduction in survival in multiple cancer types (40)	
	10 years	Extra deaths ranged from 0.0-16.6 per million women (39) 0.05-0.55% extra breast cancer, 0.28-1.18% extra cervical cancer, and 0.19-2.17% extra colorectal cancer deaths(36)	1.9-28.7%, 19.5-30.4%, and 29.7-35.0% reduction in survival for stage I, stage II, or stage III colorectal cancers (37) >30% reduction in survival in multiple cancer types (41)		0.52% extra breast cancer deaths (30)
	15 years	26% extra colorectal cancer deaths (35)			
	30 years	0.4-0.6% extra colorectal cancer deaths in NL, 1.0% extra deaths in AUS and 0.4% extra deaths in CA (31)			
	life				3.80% extra cervical cancer deaths (34)
**9 months delay**	10 years	0.11-0.83% extra breast cancer, 0.16-1.69% extra cervical cancer, and 0.27-2.55% extra colorectal cancer deaths (36)			
**12 months delay**	5 years		8.13% extra deaths of multiple cancer types (33) 4.8-16.6% extra deaths of multiple cancer types (38)		
	10 years	0.24-1.09% extra breast cancer, 0.26-2.27% extra cervical, and 0.25-3.08% extra colorectal cancer deaths (36)			
	15 years	28% extra colorectal cancer deaths (35)			
**18 months delay**	15 years	29% extra colorectal cancer deaths (35)			
**24 months delay**	15 years	30% extra colorectal cancer deaths (35)			

**Table 4:** Effect of the Covid-19 induced modifications in care on mental health.

Main author	Country	Cancer type(s)	Selection of patients	Population groups (n)	How were modifications in care determined?	Type of modifications in care	Effect on mental health
**Gultekin** (24)	Europe	Gynecological	Online and paper survey distributed among patients and survivors *via* the treating clinical team, the national patient charities and advocacy groups, social media platforms, and patients’ forums between 1st and 31st May, 2020	1251 (not mentioned how many patients had modification in care)	Patients filled in a survey	Modifications of care (of any type)	Modifications in care were associated with a higher risk of an abnormal HADS anxiety score (OR: 1.52, 95% CI: 1.07-2.16), but not with an abnormal depression score (PR: 0.75, 95% CI: 0.52-1.08).
**Joly** (25)	France	Solid and hematologic	Patients treated during the first lockdown were asked to complete a survey between 16th April and 29 May, 2020	Modifications in care: 195 No modifications in care: 539	Medical records	Delay or interruption of treatment. Change in treatment plan, method of administration, or follow-up	Modifications in care were associated with a higher risk on post-traumatic stress disorder symptoms (OR: 1.65, 95% CI: 1.03-2.63).
**Juanjuan** (26)	China	Breast	Online survey distributed among patients and survivors *via* WeChat between 16th and 19th February, 2020	Treatment alterations: 219 No treatment alterations: 255	Patients filled in a survey	Discontinue or modify treatment	Treatment alterations were associated with a higher risk of distress (p=0.046), but not with anxiety (p=0.182), depression (p=0.137), or insomnia (p=0.238).
**Kim** (27)	South Korea	Breast	Online survey distributed among patients and survivors *via* an online breast cancer community and a patient self-help social networking site between April and June, 2020	Modification in care: 29, of whom 18 experienced delays No change in treatment: 125	Patients filled in a survey	Delay or cancellation in treatment, follow-up or tests. Change in treatment plan	Delay in treatment was not associated with fear of cancer recurrence (p=0.319), anxiety (p=0.669), or depression (p=0.663). Modifications in care were not associated with fear of cancer recurrence (p=0.347), or anxiety (p=0.117), but was associated with an increased risk of depression (p=0.050).
**Xie** (28)	China	Breast	Patients referred to radiotherapy during 24th January and 30th April, 2020, for their primary or recurrent/metastatic breast cancer were asked to complete a survey between 9th and 30th April, 2020	Normal radiotherapy: 242 Delayed radiotherapy: 149 Interrupted radiotherapy: 24 Special normal (patient thought radiotherapy had been delayed, but this was not the case): 73	Patients filled in a survey	Delay or interruption of radiotherapy	Interrupted radiotherapy was associated with a higher fear-of-cancer-recurrence score (beta: 0.071, p=0.035). Delayed radiotherapy or special normal was not associated with the fear-of-cancer-recurrence score (beta: -0.01, p=0.808; beta: 0.065, p=0.06, respectively).
**Yang** (29)	China	Lymphoma	Online survey distributed among patients and survivors *via* the platform of the Chinese lymphoma patient organization between 17th and 19th April, 2020	Modifications in care: 476 No modifications in care: 570	Patients filled in a survey	Delay in therapy or exams. Less intensive therapy. Change in method of administration.	Modifications in care were not associated with the risk of anxiety (HR: 0.86, 95% CI: 0.64-1.16).

CI, confidence interval; HR, hazard ratio; OR, odds ratio.

### 3.1 Tumor characteristics

Out of the fifteen studies comparing the characteristics of tumors diagnosed during and before the COVID-19 pandemic twelve were observational studies (12–23, 31, 35, 39) and three were modelling studies (**Table 1**) (31, 35, 39). The observational studies will be discussed first.

All studies gathered their data during 2020. Three studies were nationwide population-based studies (14, 20, 22). In nine studies, the total number of tumors diagnosed during compared to before the pandemic was lower (12–15, 17, 19, 20, 22, 23). Out of these nine studies, three showed that, in percentage terms, the decrease in the number of diagnosed stage 0, I, and/or II tumors was higher than the decrease in the number of diagnosed stage III and IV tumors (14, 17, 23). In seven of the studies, tumor stage or grade distribution was different during compared to before the pandemic (13, 14, 18, 20–23), with a higher proportion of patients being diagnosed with a stage III or IV, or grade 3 tumor during the pandemic (13, 14, 18, 21–23). Out of these seven studies three studies only showed this difference in distribution in patients diagnosed in July-October, 2020, or October-December, 2020, but not in patients diagnosed earlier during the pandemic (i.e., March-June 2020 or April-June, 2020) (20, 22, 23). In one study tumor stage distribution did no longer differ between breast cancer patients diagnosed before and during the pandemic after stratification by method of detection (screen- or non-screen-detected) (14). Two studies found no difference in tumor stage distribution during compared to before the pandemic (12, 19). One study found a lower proportion of late-stage (stage III or IV) oral tumors diagnosed during the COVID-19 pandemic (16). Another study showed a higher median Brewslow thickness of cutaneous malignant melanomas diagnosed post lockdown (15).

A statistically significant higher number of people were diagnosed per month with a stage III colorectal tumor during compared to before the pandemic in the study of Kuzuu et al. (17). The study of Park et al. and Vanni et al. showed that a higher number of patients were diagnosed with a late-stage non-small-cell lung tumor or a N2, grade 2, or grade 3 breast tumor during compared to before the pandemic, respectively (18, 21). However, it was not tested whether this was statistically significant. One study found a higher number of patients diagnosed with a late-stage uveal melanoma during July-October 2020, but not during March-June 2020 (22).

Of the fifteen studies looking at tumor characteristics, three were modelling studies (31, 35, 39). One study predicted that, depending on the type of screening disruption, 0 to 1.2% of the cervical tumors will be detected at a later stage (39). The other two studies predicted that a six-month delay in screening would increase the estimated number of patients with a late-stage colorectal tumor with 18 to 33% (35), or with 1.2 to 5.7% (31).

### 3.3 Survival

Twelve modelling studies investigated the impact of delay due to COVID-19 on the survival of cancer patients (**Table 2**). Studies used different time horizons, ranging from five-year time horizons to lifetime. Five studies modelled the impact of a delay in diagnosis (32, 33, 37, 38, 41), four studies modelled delays in cancer screening (31, 35, 36, 39), two studies modelled combinations of types of delay (30, 34), and one study modelled the effect of treatment delay (40). There was also variation in the length of delay, ranging from studies using two months and other studies using up to two years of delay.

As a consequence, the effect of delay on survival showed large variation between studies. Studies estimated a 0.4 to 35% reduction in survival and a -0.04 to 30% increase in the number of deaths, depending on the type and lengths of delays. A delay of two months in diagnosis, or a combination of diagnosis and treatment was estimated to decrease survival by 0.4 to 11.5% (37), and to increase the number of cancer deaths with 2.52% (34), respectively. A delay of three months in screening, diagnosis or treatment was estimated to decrease survival by >10 to >17% (40, 41) and to increase the number of deaths by -0.04 to 1.78% (32, 36). Delays of six months in screening, diagnosis, treatment, or a combination of screening, diagnosis and treatment decreased survival with an estimated 1.9 to 35.0% and increased the number of deaths with an estimated 0.05 to 26% (30–32, 34–37, 39–41). A delay of nine months in screening was estimated to increase the number of deaths by 0.11 to 2.55% (36) A delay of twelve months in screening or diagnosis was estimated to increase the number of deaths by 0.24 to 28% (33, 35, 36, 38). In the study that modelled the longest delay, i.e., a delay of 18 and 24 months in screening, a 29% and 30% increase in the number of deaths was estimated, respectively (35).

Delay in the cancer screening programs was estimated to increase the number of deaths with -0.04 to 30% (31, 35, 36, 39). Delays in diagnosis were estimated to reduce survival with 0.4 to 35.0% and to increase the number of deaths with 0.52 to 16.6% (32, 33, 37, 38, 41). Treatment delays were estimated to be associated with a >17 to >30% lower survival (40). Delays in combinations of types of delay increased the number of estimated deaths with 0.52 to 3.80% (30, 34).

Based on the modelled time horizons in the studies, delays were estimated to decrease survival with >17 to >30% and to increase the number of deaths with 0.52 to 16.6%, over a 5-year time horizon (32, 33, 38, 40). Delays were estimated to decrease survival with 0.4 to 35.0%, and to increase the number of deaths with -0.04 to 3.08% over a ten-year time horizon (30, 36, 37, 39, 41), to increase the number of deaths with 26 to 30% over a fifteen-year time horizon (35), to increase the number of deaths with 0.4 to 1.0% over a thirty-year time horizon (31), and to increase the number of deaths with 2.52 to 3.80% over a lifetime time horizon (34).

The modelling assumptions varied between the studies. Five of the studies assumed disruptions within the United Kingdom’s urgent 2-week-wait referral pathways. The remaining seven studies each have unique assumptions. A table showcasing the estimated effects on survival per type of delay, time horizon and place within the cancer care can be found in **Table 3**.

### 3.4 Mental health

Six cross-sectional studies investigated the impact of modifications in care due to the COVID-19 pandemic on the mental health of cancer patients (**Table 4**) (24–29). Modifications in care included delays, interruptions, or cancellations of treatments, tests, or follow-up visits, and changes in the treatment plan, method of treatment administration, or follow-up. Four studies included both cancer patients and survivors (24, 25, 27, 29). Five of the studies performed their study between April to June, 2020 (24, 25, 27–29), and one in February, 2020 (26). The latter was performed in the Hubei region, the region first affected by the pandemic. In one study medical records were used to determine whether patients experienced modification in care (25), in the other five studies patients filled in a survey to detect modifications in care (24, 26–29).

Studies showed dissimilar results concerning the effects of modifications in care on the mental health of cancer patients/survivors. A positive association was seen between modifications in care and post-traumatic stress disorder symptoms (25) or levels of distress (26). One study found a positive association with fear of cancer recurrence in patients with interrupted radiotherapy, but not in patients with delayed radiotherapy (28), another study found no association with fear of cancer recurrence (27). A positive association with depression was found in one study (27), but not in two others (24, 26). One study found a positive association with anxiety (24), while no association with anxiety was found in three other studies (26, 27, 29).

## 4 Discussion

To our knowledge, this is the first systematic review that aims to give an overview of the impact of delays in screening, diagnosis and/or treatment caused by the COVID-19 pandemic on the physical and mental health outcomes of cancer patients. Overall, there was a large discrepancy between both the methods and results of the studies.

### 4.1 Tumor characteristics

Nine studies showed a decrease in the number of tumors diagnosed during compared to before the pandemic (12–15, 17, 19, 20, 22, 23). Three of these studies showed that, in percentage terms, the decrease in the number of diagnosed stage 0, I, and II tumors was higher than the decrease in the number of diagnosed stage III and IV tumors (14, 17, 23). There may be two possible complementary explanations for this larger decrease in the number of early-stage tumor diagnoses. The first may be that early-stage tumors cause mild symptoms, for which a visit to the GP might be postponed, while late-stage tumors often cause severe symptoms for which care is sought, even during a pandemic. Second, in many countries the national breast and colorectal cancer screening program was temporarily suspended during the pandemic. Tumors detected at the breast cancer screening program mainly consist of stage 0, I, or II (42). Colorectal cancer screening aims to detect pre-malignant lesions and malignancies at an early stage, mainly stage I (43). Hence, suspension of the screening program would mainly have led to a decrease in the diagnosis of adenomas and stage I tumors. The large decrease in the number of early-stage tumors causes a relative increase in the proportion of patients being diagnosed with a late-stage tumor (stage III or IV), without necessarily an actual increase in the absolute number of patients being diagnosed with a late-stage tumor.

One study showed a statistically significant increase in the number of diagnosed stage III colorectal tumors (17). However, the results of this study should be interpreted with caution as this study only included two hospitals and the other two studies including colorectal cancer patients showed a decrease in the number of patients diagnosed with a stage III tumor during the pandemic (19, 23). The results of the study of Kuzuu et al. are however in accordance with one of the two modelling studies which predicted a 25% increase in the number of late-stage tumors due to a six-month delay in screening (17, 35). Three other studies also showed an increase in the number of stage III or IV, or grade 3 tumors. However, these studies did not test whether this was significant and they only included a small number of patients (between the 146 and 223 patients diagnosed during the COVID-periods) (18, 21, 22). Therefore, these results should also be interpreted with care.

The three observational studies including colorectal cancer patients all showed a decrease in the total number of colorectal cancer diagnoses (17, 19, 23). However, this can probably not be solely attributed to the COVID-19 pandemic. Many countries implemented colorectal cancer screening programmes to detect premalignant colorectal lesions. This resulted in a decreasing trend in the number of colorectal cancer diagnoses observed in the years following the introduction of the screening programs (44).

### 4.2 Survival

Overall, the twelve included modelling studies estimated a decreased survival. However, the estimated reductions in survival varied widely. The models based their parameters on pre-COVID data, which might not be an accurate reflection of the effects caused by COVID-19 delay. The observational studies included in this review gathered data during the COVID-19 pandemic. Compared to modelling studies, observational studies are more likely to showcase a realistic effect of the pandemic with all its complexities and unique circumstances like the applied mitigation strategies and prioritizing based on urgency.

The large variance in estimates might also be explained by the differences between the models in terms of assumptions, input data and modelling choices. All of these affect the outcomes of the model, making it difficult to compare the results. It should also be noted that all models by definition have some form of selection bias due to the inherent fact that no model is built when no effect on the outcomes is expected. The historical observational data chosen for the input parameters showcased an effect between delay and survival and therefore the models reproduce that effect as well, possibly leading to a self-fulfilling prophecy. However, the outcomes of the included studies do give an indication of the range in which COVID delays might have affected survival of cancer patients.

### 4.3 Mental health

Studies included in the current review provided dissimilar results about the effect of delays and modifications in care on the mental health of cancer patients. A systematic review concluded that the COVID-19 pandemic negatively impacted the mental health of cancer patients (7). In addition, it is known that being diagnosed with cancer is an important risk factor for depression and anxiety (45). It might be possible that the COVID-19 pandemic and the diagnosis of cancer already increased the levels of depression and anxiety to such an extent that factors such as modifications in care did not further increase the levels of depression and anxiety. Moreover, good mental support and information provision by the treating clinical team might also have prevented an increase in mental health problems. However, five of the included studies did not mention whether this social support was available (24–28). One study showed that good social services were indeed associated with lower levels of anxiety (29). Finally, studies might have been too small to show an association between modifications in care and mental health.

A limitation of five of the included studies is that they distributed their survey online (26–29) or mostly online (24), thereby limiting the generalizability of the results. Furthermore, five of the included studies did not adjust for or stratified by tumor stage (24, 26–29). One study showed that patients with a late-stage tumor had higher levels of concern due to COVID-19 compared to patients with an early-stage tumor (8), suggesting that the urge of being treated timely was related to the stage. Finally, all included studies were performed at the start of the pandemic and were therefore focusing on the early onset of mental health problems, while mental health problems could disappear later in time or, conversely, develop later in time.

### 4.4 Limitations

This systematic review has several limitations. Firstly, there was large heterogeneity between the included papers (e.g. heterogeneity in cancer types, assumptions, degree of modelled delay, study period), making it difficult to observe patterns and draw conclusions. Comparability might also be hampered due to dissimilarities between countries. In addition, four of the studies on mental health did not mention what the COVID-19 induced delays entailed for patients in terms of delay or alterations in care (25–27, 29), which also complicates the comparability between studies. Secondly, we considered using a quality assessment tool to assess the quality of the included papers. However, there was a large discrepancy between the applied methods of the papers. None of the available quality assessment tools fully complied with the papers, making the overall scoring of the quality impossible. Therefore, we omitted the use of such a tool. Third, the COVID-19 pandemic is still ongoing. Long-term effects can only be predicted for now using models and historical data. The upcoming years will likely show the real outcomes of this unique period, when the follow-up period of cohorts experiencing delay will gradually increase.

## 5 Conclusion

The effect on health outcomes due to delays caused by COVID-19 remain uncertain. Observational studies describing data up till 2020 did not provide evidence for an increase in the absolute numbers and incidence of late-stage tumors, suggesting that mortality might not increase to a large extent. The modelling studies estimated that COVID-19 induced delays in screening, diagnosis, and/or treatment will lead to a lower survival of cancer patients, but the estimates varied widely and were based on selective literature showing health effects of delay. The observational studies showed varying results concerning the effect on mental health. This may be related to the fact that the studies available regarding mental health only report on a relatively short period of data collection, ranging from 3 days to 3 months. More observational studies, with a longer follow-up period, are needed to give more conclusive results about the effects of modifications in oncological care due to the COVID-19 pandemic on the physical and mental health outcomes of cancer patients. Multiple of these studies have emerged during 2022. An update of this review in the near future and a comparison of new results with our findings from 2020 and 2021 will be needed to confirm our findings, and to shed light on the role of a longer follow-up period on possible detrimental health effects of delays in cancer care.

## Data availability statement

The original contributions presented in the study are included in the article/**Supplementary Material**. Further inquiries can be directed to the corresponding author.

## Author contributions

EV and GW conceived the idea of a review article on this topic. EV and AE conducted the research and wrote the article. All authors contributed to the article and approved the submitted version.
