# Supplementary material for: Physical and mental health outcomes of COVID-19 induced delay in oncological care: A systematic review
Source: Front Oncol. 2023 Jan 27;13:998940. doi: 10.3389/fonc.2023.998940 (PMC9911660; doi:10.3389/fonc.2023.998940)
Supplement: Supplementary file 1 [file DataSheet_1.pdf]

Supplementary Material

1 Search strategy

#30	#26 NOT #27 AND ([dutch]/lim OR [english]/lim)	1,036
#29	#26 NOT #27	1,037
#28	#26 AND ('article'/it OR 'article in press'/it OR 'letter'/it OR 'review'/it) AND ([dutch]/lim OR [english]/lim)	1,333
#27	#26 AND ('article'/it OR 'article in Press'/it OR 'letter'/it OR 'review'/it)	1,362
#26	#9 AND #18 AND #24 AND [2020-2021]/py	2,399
#25	#9 AND #18 AND #24	2,496
#24	#19 OR #20 OR #21 OR #22 OR #23	254,213
#23	'corona':ti,ab AND ('patient*':ti,ab OR 'disease*':ti,ab OR 'ill*':ti,ab OR 'virus*':ti,ab)	6,387
#22	'sars-cov-2*':ti,ab OR 'sars-ncov*':ti,ab OR '2019-ncov*':ti,ab OR 'hcov-19*':ti,ab	55,620
#21	'covid-19*':ti,ab OR 'lockdown*':ti,ab OR 'pandemi*':ti,ab	207,824
#20	'2019 novel coronavirus'/exp	47,499
#19	'coronavirus*':ti,ab OR 'coronavirus disease 2019'/exp	187,478
#18	#10 OR #11 OR #12 OR #13 OR #14 OR #15 OR #16 OR #17	2,853,143
#17	'oncology'/exp/mj	124,195
#16	'oncological procedure'/exp/mj OR 'oncological parameters'/exp/mj OR 'oncol*':ti	579,792
#15	'cancer screening'/exp/mj OR 'cancer screening*':ti OR 'malignan*':ti	255,792
#14	'tumour classification'/exp OR 'tumor classificat*':ti OR 'tumour classificat*':ti OR 'tumor stag*':ti OR 'tumour stag*':ti OR 'cancer staging'/exp	491,242

#13	'cancer survival'/exp/mj OR ((cancer NEAR/2 'survival*'):ti,ab)	81,328
#12	'neoplasm'/exp/mj/dm_di,dm_dm,dm_dt,dm_rt,dm_su,dm-_th OR ('neoplasm'/exp/dm_di,dm_dm,dm_dt,dm_rt,dm_su,dm_t-h AND ('cancer*':ti OR 'neoplasm*':ti OR 'carcino*':ti OR 'oncolo*':ti))	1,756,139
#11	'cancer diagnosis'/exp/mj OR ((cancer NEAR/2 'diagno*'):ti,ab)	202,045
#10	'cancer therapy'/exp/mj OR 'cancer therap*':ti,ab OR 'cancer treatm*':ti,ab OR 'cancer care*':ti,ab OR 'cancer diagno*':ti,ab OR 'oncolo* therap*':ti,ab OR 'oncolo* treatm*':ti,ab OR 'oncolo* care*':ti,ab OR 'oncolo* diagno*':ti,ab OR 'neoplas* therap*':ti,ab OR 'neoplas* treatm*':ti,ab OR 'neoplas* care*':ti,ab OR 'neoplas* diagno*':ti,ab	537,928
#9	#1 OR #2 OR #3 OR #4 OR #5 OR #6 OR #7 OR #8	2,119,303
#8	'delaying*':ti,ab OR 'postponing*':ti,ab OR 'waiting time*':ti,ab OR 'lockdown*':ti,ab	57,446
#7	'screening'/exp AND ('delay*':ti,ab OR 'postpon*':ti,ab OR 'progression*':ti,ab OR 'recurren*':ti,ab OR 'stage*':ti,ab OR 'upstaging*':ti,ab OR 'migration*':ti,ab OR 'detection*':ti,ab OR 'avoidable':ti,ab OR 'decline*':ti,ab OR 'suspens*':ti,ab OR 'interrupt*':ti,ab OR 'deferred':ti,ab OR 'restrict*':ti,ab OR 'paused':ti,ab OR 'disrupt*':ti,ab)	175,847
#6	('therapy'/exp/mj OR 'treatment*':ti,ab OR 'care':ti,ab OR 'surgery'/exp/mj OR 'surger*':ti,ab OR 'surgical*':ti,ab) AND ('delay*':ti,ab OR 'postpon*':ti,ab OR 'progression*':ti,ab OR 'upstaging*':ti,ab OR 'migration*':ti,ab OR 'detection*':ti,ab OR 'avoidable':ti,ab OR 'decline*':ti,ab OR	1,827,300

	'suspens*':ti,ab OR 'interrupt*':ti,ab OR 'deferred':ti,ab OR 'restrict*':ti,ab OR 'paused':ti,ab OR 'disrupt*':ti,ab OR 'timing':ti,ab)	
#5	('time to treatment'/exp OR 'time to treatment*':ti,ab OR 'diagnosis time'/exp OR 'time to diagno*':ti,ab) AND ('delay*':ti,ab OR 'postpon*':ti,ab OR 'progression*':ti,ab OR 'recurren*':ti,ab OR 'stage*':ti,ab OR 'upstaging*':ti,ab OR 'migration*':ti,ab OR 'detection*':ti,ab OR 'avoidable':ti,ab OR 'decline*':ti,ab OR 'suspens*':ti,ab OR 'interrupt*':ti,ab OR 'deferred':ti,ab OR 'restrict*':ti,ab OR 'paused':ti,ab OR 'disrupt*':ti,ab)	12,363
#4	('treatment*':ti,ab OR 'therap*':ti,ab OR 'diagno*':ti,ab OR 'screening*':ti,ab OR 'diagnosis'/exp OR 'surgery'/exp OR 'surger*':ti,ab OR 'surgical*':ti,ab) AND ('delay*':ti,ab OR 'postpon*':ti,ab)	482,630
#3	'therapy delay'/exp AND ('delay*':ti,ab OR 'postpon*':ti,ab OR 'progression*':ti,ab OR 'recurren*':ti,ab OR 'stage*':ti,ab OR 'upstaging*':ti,ab OR 'migration*':ti,ab OR 'detection*':ti,ab OR 'avoidable':ti,ab OR 'decline*':ti,ab OR 'suspens*':ti,ab OR 'interrupt*':ti,ab OR 'deferred':ti,ab OR 'restrict*':ti,ab OR 'paused':ti,ab OR 'disrupt*':ti,ab) OR 'collateral damage*':ti OR 'collateral effect*':ti	12,496
#2	'delayed diagnosis'/exp OR (('delay*' NEAR/3 'diagno*'):ti,ab)	55,340
#1	'therapy delay'/exp OR (('delay*' NEAR/3 ('therap*' OR 'treatment*')):ti,ab) OR (('delay*' NEAR/3 care):ti,ab	58,738
